# Relationships among Physical Self-Concept, Physical Activity and Mediterranean Diet in Adolescents from the Province of Granada

**DOI:** 10.3390/children8100901

**Published:** 2021-10-09

**Authors:** Mariana Pérez-Mármol, Ramón Chacón-Cuberos, Eduardo García-Mármol, Manuel Castro-Sánchez

**Affiliations:** 1 Department of Research Methods and Diagnosis in Education, University of Granada, 18071 Granada, Spain; marianapm@correo.ugr.es; 2 Department of Physical Education and Sports, University of Granada, 18071 Granada, Spain; eduardogarcia@ugr.es; 3 Department of Didactics of Musical, Plastic and Corporal Expression, University of Granada, 18071 Granada, Spain; manuelcs@ugr.es

**Keywords:** psychosocial wellbeing, physical activity, diet quality

## Abstract

The aim of the present research was to analyse the relationships among physical self-concept, physical activity engagement and Mediterranean diet adherence in a sample of 1650 secondary school students from the province of Granada. The study design was descriptive–exploratory, cross-sectional and ex post facto. Measurements were taken from a single group. The PSQ, PAQ-A and KIDMED questionnaires were used to measure diet quality. Results showed the presence of a positive relationship among all dimensions of physical self-concept and physical activity engagement, with better outcomes being achieved in this self-perception with increasing engagement in sport. With regard to diet quality and its repercussions on physical self-concept, it was highlighted that the dimensions of the general self-concept, physical attractiveness and strength tended to be more positive as quality improved. In contrast, worse outcomes were produced in those with a low-quality diet. In this way, it was deemed necessary to continue investigating psychosocial factors with the aim of clarifying the relationships with psychological factors and health indicators. This would enable the development of prevention and intervention programs focused on promoting wellbeing in adolescents.

## 1. Introduction

Adolescence is an a priori term that is complex to define due to the variety of biological and sociocultural factors that intervene at the time of establishing consensus in relation to its chronology. Nonetheless, generally speaking, it defines the transitional stage located between infancy and adulthood covering, approximately, the ages of 10 y to 20 y [1]. This period is considered key in the lifecycle of human beings, given that it is during this period that the foundations of identity and personality are constructed, whilst numerous changes also take place at a physical, sexual, emotional, social and moral level. These changes later give rise to the development of variations in attitudes, thoughts, behaviours, concerns and interests, which will mark, in a decisive way, the future of adolescents [2,3]. In the search for quality of life, wellbeing and optimal development, the basic health of young people is an essential requirement. For this reason, adolescence is proposed as an excellent opportunity to promote the acquisition of healthy habits that will last throughout life [4,5,6].

Self-concept is one of the most important psychosocial factors involved in the construction and development of the “self”. This is defined as the set of opinions, hypotheses and ideas that an individual has about themselves [7]. At present, self-concept is not seen from a unidimensional or hierarchical perspective, but rather, from a multidimensional, dynamic and acquired character, which may or may not improve depending on each experience or moment. According to the model conceived of by [8,9], this construct is composed of five dimensions: social, emotional, family, academic and physical. This research focuses on the study of the last dimension mentioned, being a domain of self-concept with which it establishes high and consistent relationships [10,11]. This variable was selected because, as highlighted by numerous previous studies, a relationship exists between physical self-concept and various aspects of general health, such as physical activity engagement and correct nutrition [12,13,14,15,16,17,18], the body mass index and obesity [19,20,21]. A relationship has also been shown among social pressure, the means of communication and body dissatisfaction [22,23,24,25,26,27,28]. Specifically, physical self-concept refers to the cognitive description constructed by an individual, as a function of the following four subdomains: sport ability, physical strength, physical attractiveness and physical condition. Factors considered to influence perceptions of the aforementioned aspects concern the setting and past experiences [26,29,30].

A combination of physical activity and a balanced diet has been shown to have the best health benefits. This “holy union” can contribute to reducing the likelihood of suffering chronic conditions during adult age [31,32], perceiving greater life satisfaction and mental wellbeing [14,16,22,24,31,32,33,34,35], improving academic performance [36,37,38] and favouring variables related to the quality of life [4,15,39,40]. The need to combat physical inactivity and sedentary lifestyles has been urged at a global level in recent years. Concretely, during the current stage of the COVID-19 pandemic, being physically active and following healthy habits take on huge importance in both homes and educational institutions [41,42,43,44,45].

The infant population has reached alarming levels of a sedentary lifestyle, pointing to an increased risk of maintaining the same weight predisposition into adolescence and adulthood [46,47,48]. Given the prevalence of overweight, research in national and international contexts has been refined. It is now defined as a complex and multifaceted disease with diverse origins. Given that the influence of genetic, environmental, social and/or economic factors takes a leading role, the design of effective policy is difficult [49,50,51,52,53]. It is also notable that a relationship has been highlighted between greater body mass and a more negative physical self-concept, showing that this index is also associated with a worse image of one’s self. This reality is accentuated in adolescence given that the lack of maturity and critical thinking, together with the impact of social networks and mass media, can cause the construction of a personal identity based on beauty ideals set by society. This can pose risks to the physical and mental health of adolescents, in addition to eating disorders [54,55,56,57,58].

One of the conditioning factors behind the aforementioned incidence of overweight and high body dissatisfaction are the changing nutritional patterns that have been seen in recent years and that occur from early ages, leading to increases in the consumption of unhealthy diets (fast food). The Mediterranean diet has been confirmed to constitute one of the dietary models that is most beneficial to health due to the protective effects it exerts [59,60,61]. According to Serra [62], the Mediterranean diet is characterised by the consumption of vegetables, fruits, legumes, fish, nuts and olive oil, in addition to being low in saturated fats and rich in antioxidants. Alfonso [63] denominated this diet as the combination of foods produced by local agriculture, according to local recipes and traditional cooking methods characteristic of each geographical region. However, it also includes regular engagement in physical activity, alongside a healthy and respectful approach to the environment [64].

Another conditioning factor is the exponential increase in physical inactivity. This is issue has become so big that the WHO [65] has denominated it a general challenge to public health. Authors such as Macek et al. [66] have established minimum physical activity guidelines for children aged between 6 y and 17 y. These guidelines are as follows: engage in moderate-to-vigorous aerobic physical activity for more than 60 min a day; engage in vigorous aerobic physical activity at the least three times a week; engage in muscle strengthening activity at least three times a week. Likewise, Llamazares [67] indicated the main reasons for engaging in physical activity at these ages. These motives include benefits in the educational setting, healthier habits in general, the promotion of social relationships and the obtainment of pleasure and enjoyment. 

In this way, given the numerous research studies that have concisely related the aforementioned variables, the main objective of the present study was to determine the relationships between physical self-concept, physical activity engagement and Mediterranean diet adherence in a sample of adolescents undertaking secondary school education. Based on this objective, the following three hypotheses were proposed:

- Physical activity will be positively related to physical self-concept;
- Adolescents reporting poorer diet quality will have a lower physical self-concept;
- Physical self-concept will be strongly influenced by its dimensions, the practice of physical activity and the quality of the diet.

## 2. Materials and Methods

### 2.1. Subjects and Design

The present study was nonexperimental, quantitative, descriptive–exploratory, cross-sectional and ex post facto in nature. Measurements were taken from a single group. The study population of the present sample constituted all secondary school students enrolled at state-funded institutions in the province of Granada during the 2019/2020 academic year. According to the Statistical and Cartographic Unit housed within the Ministry of Education and Sport of the Local Government of Andalusia (Spain) (2020), students enrolled in the aforementioned general course of education in the province of Granada totalled 53,198 overall. In line with the criteria established by Otzen [68], all adolescents who agreed to participate and met certain inclusion criteria were considered as part of the study population. Inclusion criteria were as follows: (a) be enrolled in state-funded secondary education during the 2019/2020 academic year; (b) be aged between 11 y and 20 y; (c) provide signed informed consent for the handling of data pertaining to minors, where relevant. Likewise, exclusion criteria were established with the aim of obtaining a reliable sample. These were as follows: (a) suffering from any type of pathology or condition that would impede the correct completion of the questionnaire; (b) returning incomplete questionnaires or responses that could create confusion (responses crossed or rubbed out). 

**Figure 1:**
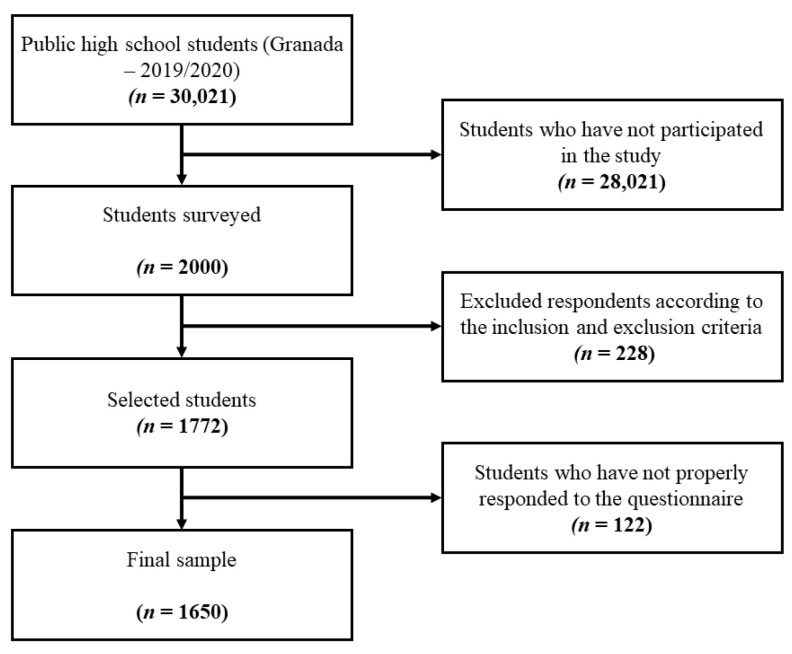
Sample distribution.

Finally, a final sample of 1650 students was obtained (Figure 1). Participants were aged between 11 y and 20 y (M = 14.48; SD = 1.41), with 50.4% (*n* = 832) being boys and 49.6% (*n* = 818) being girls. Finally, with regard to the degree of representativeness of the sample, a final sampling error of 0.023 was obtained or, in other words, a 2.37% margin of error assuming a 95% confidence level.

### 2.2. Instruments

The present study employed the following instruments: the KIDMED questionnaire. This instrument was developed by Serra [69] based on a previous study conducted with children and adolescents, denominated EnKid. Following this, the KIDMED questionnaire was conceived of, comprising 16 questions designed to evaluate the adherence of respondents to a Mediterranean diet. Responses were recorded as positive (the respondent’s diet meets dietary behaviour guidelines and corresponds to a Mediterranean diet) or negative evaluations (the respondent does not adhere to this diet). Specifically, the test was divided into four questions that had negative connotations (−1), whilst the twelve remaining questions were evaluated with a positive score (+1). The outcome was interpreted according to the following classification: (a) from 8 to 12: optimal adherence, in other words the respondent consumes a Mediterranean diet to a large extent (high); (b) from 4 to 7: moderate adherence; the respondent is required to improve his/her dietary pattern in order to adopt the Mediterranean model (medium); (c) from 0 to 3: very low-quality diet and, therefore, very low level of adherence to the Mediterranean diet (low). The reliability of the original instrument was determined as α = 0.854, and the present study obtained a lower index of α = 0.523. It should be noted that this coefficient is relatively low, which could be due to the reduced number of items and the confusion generated by the items formulated in the negative sense.

PAQ-A questionnaire: This questionnaire was developed by Martínez [70] and is composed of 9 questions, which evaluate the physical activity performed by the adolescent during the seven days prior to administration. The considered physical activities include that performed during free time, physical education classes, extracurricular classes and activity engaged in at the weekend. Questions 1 to 6 provide information about the types of sport. Questions 7 and 8 indicate physical activity intensity, alongside the frequency with which it is performed. Finally, Question 9 provides additional specific information pertaining to whether or not regular engagement in physical activity was impeded for some reason. Responses were recorded along a 5-point Likert scale. The internal consistency of this scale was α = 0.77 in the original study, with the present research obtaining a higher index of α = 0.861. The scores of this scale were worked out in two ways: (a) summation or total score of the scale; (b) division of the total score into tertiles: low level of PA (scores included in the first tertile of the total score), medium level of PA (scores included in the second tertile of the total score) and high level of PA (scores included in the third tertile of the total score). Specifically, it was observed that 34.2% (*n* = 570) of the sample were located at the low level, 50.7% (*n* = 844) at the medium level and only 14.2% (*n* = 236) associated with a high PA level.

Physical Self-concept Questionnaire (PSQ) validated by Goñi [71]: This was originally validated by these authors given the need to generate a reduced scale based exclusively on physical self-concept, in order to solve the limitations of the “*Self Description Questionnaires (SQD)*” based on the Marsh [72] model and the versions of the multidimensional self-concept of García [8]. The PSQ is a questionnaire composed of 36 items, distributed between two general scales (general physical self-concept and general self-concept) and four scales, which evaluate the four dimensions of physical self-concept (physical ability, physical condition, physical attractiveness and strength). With regard to reliability, this questionnaire achieved an excellent index of α = 0.927, with a highly similar index of α = 0.93 being obtained in a study conducted by Goñi [71]. Likewise, acceptable reliability indices were obtained for each dimension separately: GS, general self-concept (α = 0.722); SF-G, general physical self-concept (α = 0.802); SF-PA, physical ability (α = 0.754); SF-PC, physical condition (α = 0.831); SF-PAT, physical attractiveness (α = 0.803); SF-PS, physical strength (α = 0.751).

### 2.3. Procedure

Firstly, researchers from the Department of Research and Diagnostic Methods in Education, together with the Department of Musical, Artistic and Bodily Expression, put together and sent off the pertinent permissions. This was made up of informed consent forms and approval for data collection from minors. Forms were prepared for both educational institutions and families (or legal guardians). An information letter was prepared, which described the nature of the study and its aims, in addition to the research instruments that would be used and the way in which the data would be handled and used (only for scientific purposes). This serves to highlight that the present study adhered to the principles of the Declaration of Helsinki (updated in 2008) and that participants’ rights to confidentiality were respected at all times (Law 15/1999 of 13 December). Likewise, the research was supervised by the Research Ethics Committee of the University of Granada (Reference Number 2150/CEIH/2021), on 4 May 2021.

Once approval was received from educational institutions and legal guardians, data collection proceeded. Data were collected manually and in-person at the institutions, during hours agreed by the faculty and management. It should be indicated that, overall, 7 state-funded institutions from the province of Granada participated, and these data were gathered between January and March of the 2019/2020 academic year. Following the conclusion of the data collection period, data handling and analysis proceeded. As the first step, all incomplete questionnaires were discarded, along with those that presented confusing or unreliable responses. Once this task was completed, the data were cleaned in the database and introduced into the software IBM SPSS^®^ 22.0 (IBM Corp, Armonk, NY, USA) in order to create the data matrix. This process of review and transcription was performed throughout by the principal investigator with the aim of ensuring correct data handling, whilst at the same time avoiding mistakes due to data omission or incorrect assignment.

### 2.4. Data Analysis

Data analysis was conducted using the statistical package IBM SPSS^®^ 22.0 (IBM Corp, Armonk, NY, USA). Specifically, frequencies, means, standard deviations and percentages were used for the analysis of the basic descriptive data. On the other hand, associative analyses employed one-way ANOVA, the Bonferroni test for between-group associations and bivariate Pearson correlations. In order to examine the significance of associations between variables, the Pearson chi-squared test (0.05 *; 0.01 **; 0.000 ***) and Welch test were employed. Data normality was evaluated according to the values of kurtosis, with values lower than 2 being required. Homoscedasticity was examined using the Levene test. Finally, a univariate linear model was used to determine the influence of all variables on the general physical self-concept. This serves to highlight that the internal reliability of the instruments used was evaluated through Cronbach’s alpha, establishing a 95.5% reliability index.

## 3. Results

Table 1 shows the basic descriptive data of the variables under study. Specifically, it was observed that 50.4% (*n* = 832) of the subjects were men, while 49.6% (*n* = 818) were women. In relation to the tertiles established for physical activity, 34.5% (*n* = 570) showed a low level, while 51.2% (*n* = 844) had a medium level and 14.3% (*n* = 236) a high level. Diet categorization revealed that 23.3% (*n* = 385) had a low-quality diet, 49.8% (*n* = 821) of medium quality and 26.9% (*n* = 444) of high quality. Finally, the mean values of self-concept and its dimensions are shown, observing from the highest to lowest score: SF-G (22.25 ± 5.88), SF-PAT (21.34 ± 5.77), SF-PA (20.82 ± 5.20), SF-PC (20.35 ± 5.68) and SF-PS (19.21 ± 5.23). The general self-concept obtained a value of 23.28 ± 4.96.

Table 2 presents scores for physical self-concept and its dimensions according to physical activity level. In both cases, a growing and positive trend is seen with increasing sport engagement. Concretely, data obtained for the dimension describing general self-concept were higher when young people practiced more physical activity. The same pattern was seen with the dimensions pertaining to the general physical self-concept, physical ability, physical condition, physical attractiveness and strength. In fact, Table 1 shows higher mean values for each dimension of physical self-concept for the “high” category, followed by the medium and low levels.

Table 3 presents the different dimensions of physical self-concept according to diet quality. With regard to the dimension of the general self-concept, a positive trend is seen with increasing Mediterranean diet adherence, in addition to with physical attractiveness and strength. With regard to the general physical self-concept, in contrast, data revealed better outcomes in those with a poor-quality diet. The same occurred with the dimension describing physical ability, in which a negative trend was observed with a poorer-quality diet leading to better outcomes (23.07 ± 5.98 vs. 22.25 ± 5.58 vs. 19.86 ± 5.37). With regard to the dimension pertaining to physical condition, it was observed that adolescents who followed a medium-quality diet reported better values than those who consumed a low- or high-quality diet.

Table 4 presents the outcomes of the bivariate Pearson correlations conducted among the dimensions of self-concept, diet quality and physical activity levels. In relation to physical activity, a positive and direct relationship is shown with all its dimensions, with the highest correlation strength being observed for physical condition. Likewise, this is followed by physical ability, strength, general physical self-concept and physical attractiveness last.

The association between quality of the Mediterranean diet and physical self-concept is also demonstrated, with positive correlations in all cases. On this occasion, only low correlation strengths are shown, the highest being for physical condition. Moreover, the lowest correlation strength is given for the general physical self-concept and the general self-concept.

The existence of direct relationships among the general physical self-concept and its dimensions should be noted, observing a high correlation with physical attractiveness. Moderate-strength correlations are also shown in the fitness and physical ability dimensions. Finally, it is important to highlight that there is a positive and direct relationship between the quality of the diet and the level of physical activity.

Finally, a univariate linear model was carried out in order to verify the relationship of the analysed variables, considering physical self-concept as the dependent variable (Table 5). Levene’s equality test for the variances determined a value of *p* < 0.001, rejecting the null hypothesis and assuming differences among the variables. Likewise, the value of R^2^ was 0.658. This value determines a high percentage of explained variance, making the model valid to explain the dependent variable. In addition, appropriate values were obtained for the significance and effect sizes determined from the eta-squared.

The model determines how the dimensions of the physical self-concept, which act as covariates, configure the general physical self-concept. In this case, significance was obtained for physical condition and physical ability, with low effect sizes. On the other hand, physical attractiveness was the main determining variable, with a large effect size. In addition, it should be noted that physical strength was not a determining factor for physical self-concept, since significance was not observed. On the other hand, physical activity and quality of diet were introduced as independent variables. In this case, it was determined that diet did not influence physical self-concept, while physical activity had a moderate effect size.

## 4. Discussion

The purpose of the present study was to describe existing relationships among physical self-concept, physical activity engagement and adherence to the Mediterranean diet in an adolescent sample. The study arose out of the interest to better understand the influence exerted by the establishment of healthy habits, whilst also considering the aforementioned health indicators and the way in which they are linked to psychological and physical perceptions of one’s own body. The aim of this was to deepen the analysis of relevant variables and make recommendations to promote the positive development of wellbeing throughout life. In this sense, this research followed a line of high international interest as shown by various studies [13,43,73,74].

The main findings indicated that a positive relationship exists among all of the dimensions of physical self-concept and physical activity engagement, with better outcomes emerging for this dimension of self-perception with increasing sport activity. This coincides with research such as that conducted by Álvarez [75] and Fernández [13] and fulfils the first research hypothesis described above. Likewise, Revuelta [76] demonstrated that these variables share a bidirectional relationship; however, their study indicated a negative association between physical attractiveness and physical activity. In contrast, similar to what was found in the present study, Chacón [77] found a stronger correlation between physical self-concept and physical activity, with the dimension pertaining to attractiveness standing out the most. On the other hand, Muros [60] found a positive relationship between physical condition and physical activity engagement, in addition to a negative relationship between better physical condition and worse body composition. In this instance, females and students who were overweight or obese suffered from a worse physical condition. As another point, with regard to the dimension describing strength, Rodríguez [78] argued that it is a fundamental factor in the creation of children’s and adolescents’ perception of body image.

Thus, it serves to highlight the key and mediating role that is played by physical self-concept depending on the degree to which one is physically active. Along the same lines, Sánchez [79] argued the importance of this same idea, due to reciprocal influences among the physical condition, anthropometric variables and body image. To this end, Álvarez [75] emphasised the same conclusion, which is that body image influences both the physical and general self-concept, and for this reason, males show higher levels of self-concept as self-perceptions of the physical image in females tend to be harsher. Given the importance of this aspect, several authors emphasised the importance of promoting a healthy body image given the link identified among body dissatisfaction, high BMI and a negative physical self-concept, especially attractiveness [25,80,81,82].

This reality is reaffirmed by the prevalence of the two pandemics currently being faced by the world. On the one hand, global trends are seeing an increase in sedentary behaviour, higher rates of overweight/obesity and greater levels of concern about beauty ideals. On the other hand, the repercussions on general health produced by the lockdown due to COVID-19 are also being acutely perceived [83]. In this sense, despite sport being a path to improving physical, social and mental wellbeing and being one of the leisure options preferred my adolescents, the literature in general suggests that sport engagement decreases during adolescence. This is mainly because of psychological factors, such as greater stress and anxiety, academic factors, such as greater commitment to studying, and physical factors, such as weight changes [84,85,86,87]. In contrast, longitudinal studies, such as that conducted by Farooq [88], contradict the argument that this problem begins at this life stage and that females reduce their physical activity engagement substantially more than males. This being said, there can be no doubt that national and international policies are particularly important when it comes to pooling forces towards the common outcome of all adolescents meeting physical activity recommendations and, in this way, improving their wellbeing [41].

With regard to the findings obtained regarding diet quality and the consequence of this on physical self-concept, the present investigation revealed that the dimensions pertaining to the general self-concept, physical attractiveness and strength tend to be more positive as diet quality improves. In this sense, other similar studies are highlighted [89,90,91]. In contrast, with regard to the general physical self-concept and the dimension of physical ability, data revealed better outcomes in those who consumed a low-quality diet. This may be due to the interaction that takes place with social factors as this dimension tends to be associated with social events and relationships with others. Events that revolve around food such as, for example, celebrations or parties come into play here. With regard to the dimension of physical condition, adolescents consuming a medium-quality diet were observed to report better outcomes. In consideration of this, authors such as Muros [60] and Tarraga [92] also verified the existence of a correlation among the variables under discussion, namely physical activity engagement, physical condition and diet quality. In this sense, it is important to point out that the linear model carried out determined how physical strength is not a determining factor in self-concept, since this competence is not always associated with adequate levels of body composition in the adolescent population [86]. Furthermore, the quality of the diet has not been a determining factor for this variable, since the diet depends specifically on family issues in school populations [69]. Even so, the level of physical activity was relevant to configure a positive physical self-concept [30].

A number of studies have related Mediterranean diet adherence to a never-ending list of health-related variables. Tapia [93] showed that those who meet recommendations in relation to physical activity and screen time present better Mediterranean diet adherence. In a similar way, Puertas [94] revealed that this variable is directly related to physical activity engagement, hours of sleep, a better self-concept and negligible screen time. In this sense, Rosi [95] described the beneficial relationship of adherence to sleeping habits. Onetti [96], on the other hand, discussed the positive relationship of diet with academic and social self-concept, age and educational level. Finally, Carrillo [97] and Meyer [98] highlighted the role of weight as a moderating factor of diet quality, health perceptions and happiness.

Finally, it is important to point out some of the limitations associated with this study. In the first place, it should be noted that many of the factors studied are related to the body mass index (BMI), which was not considered in this research. Therefore, it would be of relevant interest to consider this dependent variable in future studies. Second, the nature of the research should be highlighted, as it is a nonexperimental study. This forces us to interpret the data with caution, since causal relationships cannot be determined. Finally, it is worth noting the interest of expanding the study sample considering data reported by the families of young adolescents. This is because, during this school stage, young people live with their families, which have a great influence on the healthy habits they develop.

In conclusion, the multiple benefits of incorporating healthy behaviours such as physical activity and maintaining a Mediterranean diet in adolescence are emphasized. However, it is also considered important to warn about the consequences of excessive physical exercise, consuming an overly restrictive diet or holding strong negative body image perceptions, outlining the various potential alterations to physical–mental health, such as the development of eating disorders. In this way, the need is highlighted to further investigate psychological factors with the aim of clarifying the relationships among psychological factors and health indicators. This will enable better development of prevention and intervention programs focused on promoting wellbeing in adolescents. To this end, it is important to equip students with skills that integrate psychological (reinforcing self-esteem and self-concept, reinforcing diversity and body image acceptance), social (social skills), general health (nutritional education, guidelines for physical activity, sleeping habits and the prevention of harmful behaviour) and educational (studying techniques, stress management) tools.

## Figures and Tables

**Table 1:** Descriptive data of the sample.

		**Frequency (*n*)**	**Percentage (%)**
Gender	Men	832	50.4
	Women	818	49.6
Tertiles of physical activity	Low	570	34.5
	Medium	844	51.2
	High	236	14.3
Tertiles of diet quality	Low	385	23.3
	Medium	821	49.8
	High	444	26.9
		**Mean Score**	**Standard Deviation (SD)**
Self-concept	SF-PA	20.82	5.20
	SF-PC	20.35	5.68
	SF-PAT	21.34	5.77
	SF-PS	19.21	5.23
	SF-G	22.25	5.88
	GS	23.28	4.96

Note: GS, general self-concept; SF-G, general physical self-concept; SF-PA, physical ability; SF-PC, physical condition; SF-PAT, physical attractiveness; SF-PS, physical strength.

**Table 2:** The levels of physical self-concept according to the levels of physical activity.

	PA Level	M	SD	95% CI		Homogeneity of Variance		ANOVA		Welch Test	
				LL	UL	Lev.	Sig.	F	Sig.	W	Sig.
GS	Low	22.29 *^a,b,c^*	5.09	21.87	22.71	1.06	0.344	19.96	0.000	-	-
	Medium	23.65 *^a,b^*	4.82	23.32	23.97						
	High	24.38 *^a,c^*	4.78	23.77	24.99						
SF-G	Low	20.48 *^a,b,c^*	6.10	19.98	20.98	4.21	0.015	46.06	0.000	43.16	0.000
	Medium	22.91 *^a,b,c^*	5.47	22.53	23.28						
	High	24.19 *^a,b,c^*	5.72	23.46	24.92						
SF-PA	Low	17.69 *^a,b,c^*	4.91	17.29	18.10	1.90	0.149	222.60	0.000	-	-
	Medium	21.96 *^a,b,c^*	4.52	21.65	22.26						
	High	24.30 *^a,b,c^*	4.22	23.75	24.84						
SF-PC	Low	16.89 *^a,b,c^*	5.09	16.47	17.31	1.80	0.165	251.07	0.000	-	-
	Medium	21.45 *^a,b,c^*	5.03	21.11	21.79						
	High	24.77 *^a,b,c^*	4.46	24.19	25.34						
SF-PAT	Low	19.80 *^a,b,c^*	6.14	19.29	20.30	9.39	0.000	41.14	0.000	40.93	0.000
	Medium	21.78 *^a,b,c^*	5.41	21.41	22.15						
	High	23.49 *^a,b,c^*	5.10	22.84	24.15						
SF-PS	Low	16.82 *^a,b,c^*	4.85	16.42	17.22	0.11	0.891	139.65	0.000	-	-
	Medium	19.83 *^a,b,c^*	4.87	19.50	20.16						
	High	22.77 *^a,b,c^*	4.68	22.17	23.37						

Note 1: GS, general self-concept; SF-G, general physical self-concept; SF-PA, physical ability; SF-PC, physical condition; SF-PAT, physical attractiveness; SF-PS, physical strength. Note 2: *^a, b, c,^* Post hoc (Bonferroni)—pairwise between-group comparisons. Note 2: PA, Physical Activity; M, Mean; SD, Standard Deviation; CI, Confidence Intervals; LL, Lower Limit; UL, Upper Limit; Lev., Levene-test; Sig., Level of significance; F, F-test; W, Welch statistic.

**Table 3:** Levels of physical self-concept according to diet quality.

	Diet Quality	M	SD	95% CI		Homogeneity of Variance		ANOVA	
				LL	UL	Lev.	Sig.	F	Sig.
GS	Low	22.46 *^a,b,c^*	5.01	21.95	22.96	0.24	0.787	10.40	0.000
	Medium	23.27 *^a,b,c^*	4.94	22.93	23.61				
	High	24.03 *^a,b c,^*	4.86	23.57	24.48				
SF-G	Low	23.28 *^c^*	4.96	23.04	23.52	0.69	0.497	8.54	0.000
	Medium	21.38 *^b,c^*	5.95	20.79	21.98				
	High	22.22 *^a,b,c^*	5.75	21.82	22.61				
SF-PA	Low	23.07 *^a,b,c^*	5.98	22.51	23.63	1.80	0.165	18.24	0.000
	Medium	22.25 *^a,b,c^*	5.88	21.97	22.54				
	High	19.86 *^a,b,c^*	5.37	19.32	20.40				
SF-PC	Low	20.64 *^a,b,c^*	5.19	20.29	21.00	0.18	0.834	25.24	0.000
	Medium	21.97 *^a,b,c^*	4.87	21.52	22.43				
	High	20.82 *^a,b,c^*	5.20	20.57	21.07				
SF-PAT	Low	19.04 *^a,b,c^*	5.55	18.48	19.59	1.14	0.319	11.38	0.000
	Medium	20.19 *^a,b,c^*	5.61	19.81	20.58				
	High	21.77 *^a,b,c^*	5.63	21.25	22.30				
SF-PS	Low	20.35 *^a,c^*	5.68	20.07	20.62	0.04	0.960	13.77	0.000
	Medium	20.40 *^b,c^*	5.96	19.80	21.00				
	High	21.26 *^a,b,c^*	5.61	20.88	21.65				

Note 1: GS, general self-concept; SF-G, general physical self-concept; SF-PA, physical ability; SF-PC, physical condition; SF-PAT, physical attractiveness; SF-PS, physical strength. Note 2: *^a, b, c,^* Post hoc (Bonferroni)—pairwise between-group comparisons. Note 2: M, Mean; SD, Standard Deviation; CI, Confidence Intervals; LL, Lower Limit; UL, Upper Limit; Lev., Levene-test; Sig., Level of significance; F, F-test.

**Table 4:** Bivariate Pearson correlations of physical self-concept with diet quality and physical activity.

	SF-PC	SF-PAT	SF-PS	SF-G	GS	DIET	PA
SF-PA	0.703 **	0.398 **	0.581 **	0.421 **	0.362 **	0.153 **	0.492 **
SF-PC		0.496 **	0.552 **	0.503 **	0.382 **	0.189 **	0.528 **
SF-PAT			0.364 **	0.797 **	0.644 **	0.118 **	0.231 **
SF-PS				0.362 **	0.246 **	0.127 **	0.414 **
SF-G					0.643 **	0.099 **	0.248 **
GS						0.126 **	0.163 **
DIET							0.230 **

Note 1: ** = *p* < 0.01; Note 2: GS, general self-concept; SF-G, general physical self-concept; SF-PA, physical ability; SF-PC, physical condition; SF-PAT, physical attractiveness; SF-PS, physical strength; PA, Physical Activity.

**Table 5:** Univariate linear model for physical self-concept.

Origin	Sum of Squares	DF	MS	F	Sig.	η²
Corrected model	39,440.54	153	257.78	21.70	0.000	0.689
Intersection	29,700	1	297.00	25.00	0.000	0.016
SF-PA	8881	1	88.81	7.47	0.006	0.005
SF-PC	22,078	1	220.78	18.59	0.000	0.012
SF-PAT	19,775.48	1	19,775.48	1665.16	0.000	0.527
SF-PS	666	1	6.66	0.56	0.454	0.000
PA	1022.27	52	19.65	1.65	0.003	0.054
Diet	3629	2	18.14	1.52	0.217	0.002
PA ∗ Diet	1091.71	95	11.49	0.96	0.569	0.058
Error	17,766.50	1496	11.87			
Total corrected	57,207.04	1649				

Note 1: DF, Degrees of Freedoom; PA, Physical Activity; MS, Mean Square.

## Data Availability

The data presented in this study are available on request from the corresponding author.
